# Orage rythmique chez un patient porteur d’une cardiomyopathie dilatée et un défibrillateur automatique implantable (DAI)

**DOI:** 10.11604/pamj.2017.27.31.12274

**Published:** 2017-05-11

**Authors:** Stéphane Méo Ikama, Jospin Makani, Bertrand Ellenga-Mbolla, Louis Igor Ondze-Kafata, Thierry Raoul Gombet, Gisèle Kimbally-Kaky

**Affiliations:** 1 Service de Cardiologie, CHU de Brazzaville, Congo; 2 Service des Urgences, CHU de Brazzaville, Congo

**Keywords:** Arythmie ventriculaire, cardiomyopathie dilatée, DAI, Congo, Ventricular arrhythmia, dilated cardiomyopathy, AID, Congo

## Abstract

Les arythmies ventriculaires graves sont fréquentes au cours de l’insuffisance cardiaque, mettant en jeu le pronostic vital du fait du risque accru de mort subite. Leur prise en charge efficace reste limitée en Afrique Subsaharienne, du fait des moyens limités ou non disponibles comme le défibrillateur automatique implantable (DAI). Nous rapportons l’observation d’un patient de 56 ans, porteur d’une cardiomyopathie dilatée non ischémique à fraction d’éjection du ventricule gauche (FEVG) très abaissée, et qui a bénéficié en 2012 de l’implantation d’un DAI en prévention primaire de mort subite pour des arythmies ventriculaires. Le traitement d’entretien associait un diurétique, un IEC, et un anti-vitamine K. Le patient a présenté au mois de novembre 2014 des épisodes itératifs de décharges électriques délivrées par le DAI, sans sensation de palpitations suggestives d’épisodes d’arythmies. L’examen clinique est pauvre, en particulier pas de signes d’insuffisance cardiaque. L’interrogation du DAI a objectivé de nombreux épisodes de tachycardie et fibrillation ventriculaires ayant justifié le traitement par ATP ou par chocs de 15 joules. Le patient est mis sous amiodarone et bêtabloquant. L’évolution a été favorable avec un recul de trois mois, marquée par la reprise d’une vie normale, sans nouvel épisode de choc. Les anti-arythmiques gardent une importance capitale en cas d’arythmies ventriculaires graves, même en présence d’un DAI.

## Abstract (en)

Severe ventricular arrhythmias are frequent during heart failure; they are a life-threatening condition due to the increased risk of sudden death. Efficient management remains limited in sub-Saharan Africa because of the limited or unavailable medical resources as automated implantable defibrillator (AID). We report the case of a 56-year old patient with non ischemic dilated cardiomyopathy with very low left ventricular ejection fraction (LVEF)who underwent AID implantation for primary prevention of sudden cardiac death due to ventricular arrhythmias in 2012. Maintenance therapy combined diuretic, angiotensin-converting enzyme (ACE) inhibitor and anti-vitamin K. In the month of November 2014 the patient had iterative episodes requiring the delivery of electric shocks by the AID, without the sensation of palpitations suggestive of episodes of arrhythmias. Clinical examination is a poor screening test, especially for heart failure. AID detected multiple episodes of tachycardia and ventricular fibrillation justifying antitachycardia pacing (ATP) therapy or the delivery of electric shocks of 15J. The patient was treated with amiodarone and beta blocker. Evolution was favorable at 3-months follow-up. The patients had resumed normal activities, without experiencing new episodes requiring the delivery of electric shocks. This study emphasizes the essential role of anti-arrhythmic drug therapy for severe ventricular arrhythmias, even in the presence of AID.

## Introduction

Les arythmies ventriculaires graves sont fréquentes au cours de l’insuffisance cardiaque, mettant en jeu le pronostic vital du fait du risque accru de mort subite [1, 2]. La prévention de la mort subite d’origine rythmique chez ces patients constitue un challenge en Cardiologie [3]. La prise en charge efficace de ces arythmies reste difficile en Afrique Subsaharienne, du fait d’une part des moyens limités ou non disponibles comme le défibrillateur automatique implantable (DAI), et d’autre part des conditions de vie souvent modestes des populations. Nous rapportons un cas d’orage rythmique chez un patient porteur d’un DAI.

## Patient et observation

Un homme de 56 ans, chauffeur de profession, se présentait à notre consultation pour avis cardiologique. Il est connu comme porteur d’une cardiomyopathie dilaté (CMD) à coronaires saines, avec fraction d’éjection abaissée, ayant présenté plusieurs épisodes d’insuffisance cardiaque. Ce dernier a bénéficié en 2012 de l’implantation d’un DAI monochambre de marque Saint Jude Médical, pour des arythmies ventriculaires. Depuis un mois (novembre 2014), il signale des épisodes itératifs de décharges électriques délivrées par le DAI, épisodes inquiétants, angoissants, sans sensation de palpitations. Dans ses antécédents, on ne note rien de particulier, et il ne présente aucun facteur de risque cardiovasculaire. Le traitement d’entretien associe du furosémide per os, du périndopril, et de l’acénocoumarol. L’examen clinique du 05/12/2015 notait un patient en surpoids (IMC = 29,7 kg/m^2^), paucisymptomatique en classe fonctionnelle NYHA I-II, apyrétique. La pression artérielle était symétrique à 107/68 mmHg, pour une fréquence cardiaque à 108 bpm. Les bruits du cœur étaient assez réguliers, entrecoupés de quelques extrasystoles, sans signe d’insuffisance cardiaque. Les pouls périphériques proximaux et distaux étaient présents et symétriques. L’auscultation pleuropulmonaire était normale.

**Figure 1:**
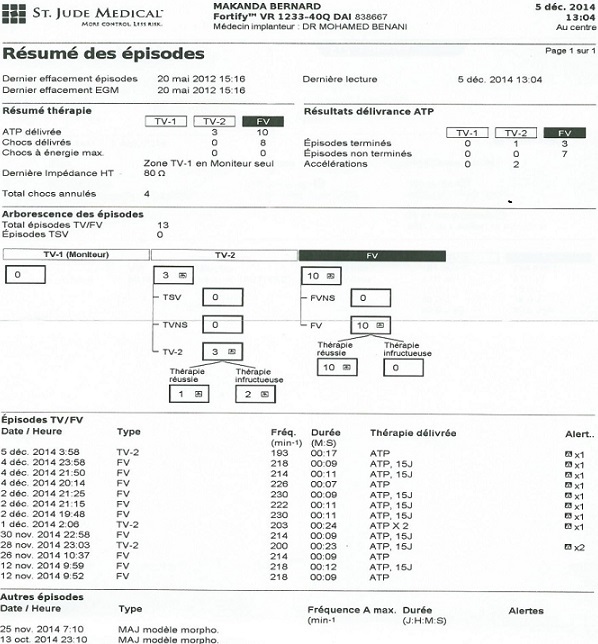
Rapport d’interrogation du DAI objectivant plusieurs épisodes d’arythmies ventriculaires (tachycardie et fibrillation ventriculaires) et les traitements délivrés sous forme d’ATP ou de chocs de 15 joules

**Figure 2:**
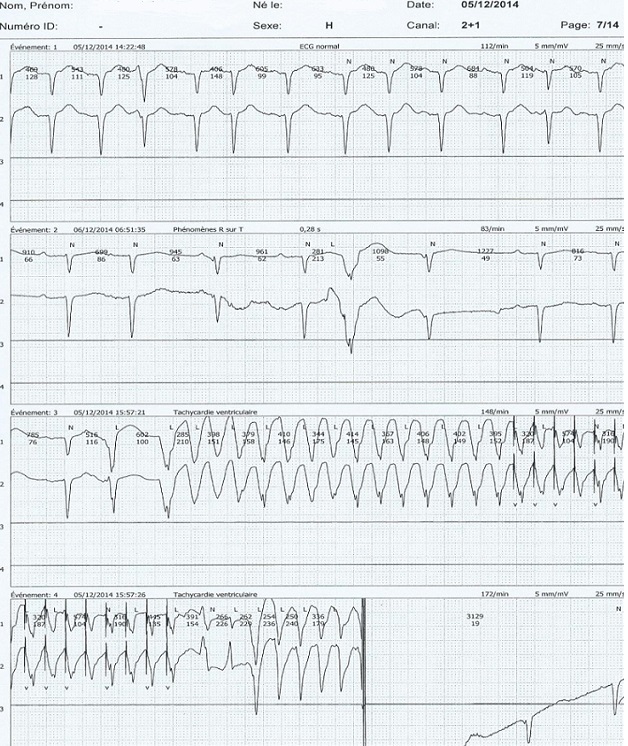
Holter ECG des 24h, mettant en évidence des troubles d’excitabilité ventriculaires et un épisode de tachycardie ventriculaire soutenue

L’électrocardiogramme de repos s’inscrivait en rythme sinusale, FC = 111 cpm, aspect de bloc incomplet gauche, avec des extrasystoles ventriculaires isolées. L’interrogation du DAI par un programmateur MERLIN (Figure 1) a objectivé 12 épisodes d’arythmies ventriculaires à type de tachycardie et de fibrillation ventriculaires, ayant nécessité des traitements par ATP (antitachycardiapacing) et par des chocs de 15 joules. Devant cette urgence, l’adjonction d’un traitement anti-arythmique a été initiée, associant de l’amiodarone per os à la dose de charge, et un bêtabloquant. L’échocardiographie transthoracique a mis en évidence un aspect de CMD hypokinétique avec altération de la fonction systolique du VG (FE = 30%). Le Holter ECG des 24 heures (Figure 2) a objectivé de nombreuses extrasystoles ventriculaires, parfois bigéminées, avec un épisode de tachycardie ventriculaire soutenue, ayant nécessité un choc signalé par la patient. L’évolution à H24 était assez favorable, le patient décrivant un léger mieux, avec une première nuit calme depuis plusieurs jours. Nous avions recommandé la poursuite du traitement associant furosémide, périndopril, amiodarone, nébivolol, et acénocoumarol. Le patient a été revu un mois après, signalant un mieux être et une reprise d’activités professionnelles. L’interrogation du DAI n’a pas objectivé d’événements rythmiques récents, et le Holter ECG dans les limites de la normale. Le traitement en cours a été reconduit et optimisé, la condition clinique et hémodynamique du patient restant stable avec un recul de trois mois.

## Discussion

La survenue d’arythmies ventriculaires malignes marque un tournant majeur dans l’évolution du patient insuffisant cardiaque, et constitue un facteur de mauvais pronostic au cours de la cardiomyopathie dilatée ischémique ou non [1, 2]. L’évaluation et la stratification du risque rythmique représentent une étape capitale dans leur prise en charge afin d’identifier les stratégies de prévention de la mort subite [3]. L’incidence de la mort subite est accrue au cours de la CMD aussi bien ischémique que non ischémique [2]. La mort subite est l’un des modes de décès dans l’insuffisance cardiaque [4], le plus souvent chez des patients en classe fonctionnelle NYHA II et III, et sa prévention constitue un challenge en cardiologie, en particulier chez des patients porteurs d’une cardiopathie et dysfonction systolique ventriculaire gauche, avec ou sans insuffisance cardiaque [3]. Deux approches majeures, souvent complémentaires, sont recommandées pour cette prévention: l’approche médicamenteuse, basée sur l’utilisation des antiarythmiques (amiodarone, avec ou sans bêtabloquant), et l’approche instrumentale basée sur l’implantation d’un défibrillateur automatique implantable (DAI) [3–6].

Concernant les antitarythmiques, ceux de la classe III de la classification de Vaughan Williams (amiodarone, sotalol, dofétilide, azimilide) sont particulièrement recommandés car ils ont des effets bénéfiques sur les fréquents chocs induits par les DAI chez des patients qui en sont porteurs [7, 8], tel était le cas de notre patient. Aussi, les antiarythmiquessont considérés comme nécessaires chez plus de 70% des patients porteurs de DAI pour plusieurs raisons [7]: ils permettent de prévenir et traiter les épisodes de tachyarythmies atriales (la fibrillation atriale étant l’arythmie la plus fréquente, survenant chez plus de 20% de patients porteurs d’un DAI) [7]; les antiarythmiques contribuent à diminuer la fréquence des chocs des DAI; enfin, ils permettent de juguler les arythmies ventriculaires résultant des traitements par stimulation antitachycardique (ATP) délivrés par les DAI.

Le DAI peut être recommandé aussi bien en prévention primaire que secondaire. D’après les recommandations de la Société Européenne de Cardiologie (ESC) de 2008et 2010 [9, 10], le DAI est recommandé en prévention secondaire chez les patients ayant survécu à une fibrillation ventriculaire ainsi que chez les patients ayant des tachycardies ventriculaires hémodynamiquement mal tolérées et/ou des tachycardies ventriculaires syncopales documentées, une fraction d’éjection du ventricule gauche (FEVG)≤40%, sous traitement médical optimal et dont l’espérance de vie en bon état fonctionnel est supérieure à un an [9]. Le DAI est recommandé en prévention primaire pour réduire la mortalité chez les patients présentant une dysfonction ventriculaire gauche due à un antécédent d’infarctus du myocarde (IDM), au moins 40 jours après l’IDM, avec une FEVG≤35%, en classe fonctionnelle NYHA II ou III, sous traitement médical optimal, et dont l’espérance de vie en bon état fonctionnel est supérieure à un an [9]. Enfin, le DAI est recommandé en prévention primaire pour réduire la mortalité chez les patients porteurs d’une myocardiopathie non ischémique avec une FEVG≤35%, en classe fonctionnelle NYHA II ou III, sous traitement médical optimal, et dont l’espérance de vie en bon état fonctionnel est supérieure à un an [9], tel était le cas de notre patient.

Par ailleurs, les bénéfices du DAI en prévention primaire ou secondaire sont sous-tendus par plusieurs grandes études [11–19]. En prévention secondaire [11], le DAI a permis une réduction notable de la mortalité aussi bien globale (-28%) que rythmique (-50%), chez des patients porteurs d’une cardiomyopathie ischémique ou non, avec un bénéfice considérable chez ceux ayant une dysfonction systolique sévère (FEVG ≤ 35%). En prévention primaire, si le bénéfice a été net dans les cardiomyopathies ischémiques (MADIT, MUSTT, MADIT II, DINAMIT) [12–15], les premiers essais n’ont pas été bénéfiques dans les cardiomyopathies non ischémiques (CAT, AMIOVIRT, DEFINITE) [16–18]. C’est grâce à l’étude SCD-HeFT [19] que le bénéfice du DAI en prévention primaire dans les cardiomyopathies non ischémiques a été évident, avec une réduction du taux de mortalité de l’ordre de -23%. Pour notre patient, en l’absence du DAI, le décès par mort subite aurait été la modalité évolutive de sa CMD non ischémique.

## Conclusion

Les arythmies ventriculaires sont fréquentes au cours des cardiomyopathies dilatées, cause fréquente d’insuffisance cardiaque en Afrique subsaharienne, et de mauvais pronostic du fait du risque accru de mort subite. Leur prévention par les traitements antiarythmiques et/ou le défibrillateur automatique implantable (DAI) constitue un challenge en cardiologie. Notre cas clinique met en exergue la nécessité d’un traitement optimal de l’insuffisance cardiaque associant diurétiques de l’anse, bloqueurs du système rénine-angiotensine, bêtabloquants, antagonistes des récepteurs des minéralocorticoïdes, mais aussi les aintiarythmiques chez des patients présentant un risque rythmique élevé, porteurs ou non d’un DAI.

## Conflits d’intérêts

Les auteurs ne déclarent aucun conflit d'intérêt.

## References

[1] Ansalone G, Giannantoni P, Santini M (1998). The stratification and prevention of the arrhythmia risk in nonischemic dilated cardiomyopathy. G Ital Cardiol..

[2] Lacoviello M, Monitillo F (2014). Non-invasive evaluation of arrhythmic risk in dilated cardiomyopathy: from imaging to electrocardiographic measures. Wolrd J Cardiol..

[3] Klein H, Auricchio A, Reek S, Geller C (1999). New primary prevention trials of sudden cardiac death in patients with left ventricular dysfunction: SCD-Heft and MADIT-II. Am J Cardiol..

[4] MERIT-HF Investigators (1999). Effect of metoprolol CR/XL in chronic heart failure: Metoprolol CR/XL Randomized Intervention Trial in Congestive Heart Failure (MERIT-HF). Lancet..

[5] (2006). ACC/AHA/ESC 2006 guidelines for management of patients with ventricular arrhythmias and the prevention of sudden cardiac death: A report of the American College of Cardiology/American Heart Association Task Force and the European Society of Cardiology Committee for Practice Guidelines (Writing Committee to Develop Guidelines for Management of Patients with Ventricular Arrhythmias and the Prevention of Sudden Cardiac Death). Developed in collaboration with the European Heart Rhythm Association and the Heart Rhythm Society. Europace..

[6] (2006). ACC/AHA/ESC 2006 guidelines for management of patients with ventricular arrhythmias and the prevention of sudden cardiac death-executive summary. A report of the American College of Cardiology/American Heart Association Task Force and the European Society of Cardiology Committee for Practice Guidelines (Writing Committee to Develop Guidelines for Management of patients with Ventricular Arrhythmias and the Prevention of Sudden Cardiac Death). Developed in collaboration with the European Heart Rhythm Association and the Heart Rhythm Society. Eur Heart J..

[7] Bollmann A, Husser D, Cannom DS (2005). Antiarrhythmic drugs in patients with implantable cardioverter-defibrillators. Am J Cardiovasc Drugs..

[8] Karaoguz R, Maydanozcu S, Altun T, Güldal M, Akyürek O, Erol C (2006). Appropriate ICD therapy in patients with idiopathic dilated cardiomyopathy: long term follow-up. Int Heart J..

[9] (2008). ESC Guidelines for the diagnosis and treatment of acute and chronic heart failure 2008: the Task Force for the Diagnosis and Treatment of Acute and Chronic Heart Failure 2008 of the European Society of Cardiology. Developed in collaboration with the Heart Failure Association of the ESC (HFA), and endorsed by the European Society of Intensive Care Medicine (ESICM). Eur Heart J..

[10] (2010). 2010 Focused Update of ESC Guidelines on device therapy in heart failure. An update of the 2008 ESC Guidelines for the diagnosis and treatment of acute and chronic heart failure and the 2007 ESC guidelines for cardiac and resynchronization therapy - Developed with the special contribution of the Heart Failure Association and the European Heart Rhythm Association. Eur Heart J..

[11] Connoly SJ, Hallstrom AP, Cappato R (2000). Meta-analysis of the implantable cardioverter defibrillator secondary prevention trials - AVID, CSAH and CIDS studies.Antiarrhytymicvs implantable defibrillator study - Cardiac Arrest Study Hamburg. Canadian Implantable Defibrillator Study. Eur Heart J..

[12] Moss A, Hall J, Cannom D (1996). Improved survival with an implantable defibrillator in patients with cororary disease at high risk for ventricular arrhythmia - The MADIT trial. N Engl J Med..

[13] Buxton A, Lee K, DiCarlo L (2000). Electrophysiologic testing to identify patients with coronary artery disease who are at risk for sudden cardiac death. The MUSTT trial. N Engl J Med..

[14] Moss AJ, Zareba W, Hall J (2002). Prophylactic implantation of a defibrillator in patients with myocardial infarction and reduced ejection fraction. N Engl J Med..

[15] Honhloser S, Kuck H, Dorian P (2004). Prophylactic use of an implantable cardioverter-defibrillator after acute myocardial infarction. N Engl J Med..

[16] Bansch D, Antz M, Boczor S (2002). Primary prevention of sudden cardiac death in idiopathic dilated cardiomyopathy. Circulation..

[17] Strickberger S, Hummel J, Bartlett T (2003). Amiodarone versus implantable cardioverter-defibrillator: randomized trial in patients with non ischemic dilated cardiomyopathy and asymptomatic nonsustained ventricular tachycardia-AMIOVIRT. J Am Coll Cardiol..

[18] Kadish A, Dyer A, Daubert P (2004). Prophylactic defibrillator implantation in patients with non ischemic dilated cardiomyopathy. N Engl J Med.

[19] Bardy GH, Lee KL, Mark DB (2005). Sudden Cardiac Death in Heart Failure Trial (SCD-HeFT) investigators - Amiodarone or an implantable cardioverter-defibrillator for congestive heart failure. N Engl J Med..

